# Anti-Mullerian Hormone Gene Polymorphism in Polycystic ovary syndrome: A pilot study

**DOI:** 10.12669/pjms.41.9.12104

**Published:** 2025-09

**Authors:** Laiba Rehmat, Samar Zaki, Haq Nawaz Khan, Unab I Khan, Rehana Rehman

**Affiliations:** 1 Laiba Rehmat, MPhil-BBS Graduate Student; 2 Samar Zaki Assistant Professor, Department of Family Medicine, The Aga Khan University, Stadium Road Karachi, Pakistan; 3 Haq Nawaz Khan Senior Instructor, Department of Pathology & Laboratory Medicine, The Aga Khan University, Stadium Road Karachi, Pakistan; 4 Unab I. Khan Professor, Department of Family Medicine, The Aga Khan University, Stadium Road Karachi, Pakistan; 5 Rehana Rehman Professor, Biological and Biomedical Sciences, The Aga Khan University, Stadium Road Karachi, Pakistan

**Keywords:** Anti-Mullerian hormone, *AMH*, Genetic variants, Polycystic ovary syndrome, Reaction-Restricted Fragment Length Polymorphism, Sanger sequencing

## Abstract

**Background & Objective:**

Polycystic ovary syndrome (PCOS) is a heterogeneous endocrine condition; characterized by ovulatory disturbance, which is attributed to elevated anti Mullerian hormone (AMH) levels. We compared AMH levels in females with and without PCOS and aimed to determine the genetic variant; rs10407022 in *AMH* of Pakistani females with PCOS.

**Methodology:**

This case control study was conducted at Aga Khan University from January, 2024 till November, 2024 on 99 female subjects (49 PCOS, 50 controls). Serum AMH levels were measured using commercially available kits and the genotyping of *AMH* rs10407022 SNP was done by Tetra-ARMS-PCR in both groups. Results were validated by Polymerase Chain Reaction-Restricted Fragment Length Polymorphism (PCR-RFLP). Final validation was conducted via Sanger DNA sequencing of randomly selected samples of PCOS females.

**Results:**

Higher AMH levels were observed in females with PCOS [6.47 ± 6.05 vs 3.06 ± 1.91(ng/ml); *p* value <0.0001]. PCR amplified the AMH (rs10407022) in PCOS samples, showing control band at 238 bp and band at 135 bp for genotype. Further, the SNP (Homozygous WT/MT and heterozygous) was validated by RFLP and a gold standard Sanger DNA sequencing.

**Conclusion:**

High AMH levels associated with the genetic variant (rs10407022) of *AMH* suggest role of *AMH* gene dysregulation in the manifestation of PCOS.

## INTRODUCTION

Polycystic ovary syndrome (PCOS) is a heterogeneous endocrine condition. The diagnostic criterion requires presence of at least two of the following three features: ovulatory disturbance, clinical and/or biochemical hyper androgenism, and polycystic ovarian morphology.^1^,^2^ This endocrine disorder is common globally among women of reproductive age, with a prevalence ranging from 5% to 15%.^3^,^4^ In Pakistani women, the prevalence is significantly higher, reaching 52%.^5^

In addition to the three primary diagnostic features, elevated serum levels of anti-Müllerian hormone (AMH) has been observed in females with PCOS.^6^-^8^ AMH is a member of the transforming growth factor beta family and is secreted by the granulosa cells of growing, gonadotropin-independent follicles, particularly the pre-antral and small antral follicles.^9^ Serum AMH concentration is proportional to the number of developing follicles in the ovaries.^10^ Within the ovary, AMH plays a crucial role in follicular development and the selection of the dominant follicle during which follicular sensitivity to FSH is increased. AMH inhibits this FSH-dependent follicular growth contributing to the accumulation of small antral follicles and ovulatory disturbance in PCOS.^10^ AMH levels are found to be the strongest diagnostic marker in patients with PCOS,^11^,^12^ potentially surpassing follicle count as an assessment tool^13^,^14^ and levels greater than 3.8–5 ng/mL are suggestive of PCOS.^11^

With reference to genetic polymorphism, a variant (rs10407022) due to a change from isoleucine to serine in AMH protein gene, affects the hormone’s biological activity. This variation influences several outcomes during Assisted Reproductive Techniques (ART), including the number of retrieved oocytes, duration of stimulation, gonadotropin dosage, and pregnancy rates.^15^ Despite the known role of *AMH i*n menstrual disorders and fertility, no significant association was found between the rs10407022 SNP in *AMH* and the reproductive outcomes of PCOS in the Gujarati population.^16^ However, the South Asian population is highly diverse, with various ethnic groups exhibiting distinct genetic predisposition to diseases, likely due to differences in their ancestral backgrounds.^17^ This genetic variation may influence the expression and impact of specific polymorphisms across populations. Such heterogeneity highlights the need for further research to better understand the role of AMH polymorphisms in different ethnic and geographic groups. Investigating this association in the genetically heterogeneous Pakistani female population may help to determine how AMH variants impact PCOS risk in specific ethnic populations.

Therefore, we aimed to compare AMH levels between PCOS cases and healthy controls and investigate the association of the rs10407022 variant in the AMH gene with PCOS in Pakistani females. This study will help to address a gap in existing research and enhance understanding of the genetic factors contributing to the manifestation of PCOS in our population. Identifying these factors may aid in developing preventive and predictive strategies for diagnosis and treatment of females with PCOS.

## METHODOLOGY

This case control study was conducted in Aga Khan University from January, 2024 till November, 2024. Females diagnosed with PCOS, aged 18-45 years meeting at least two of the following criteria: increased androgen activity, polycystic ovaries on ultrasound examination and/ or oligo/anovulation were recruited as cases. The control group comprised of females without features of PCOS (age and BMI matched to the best). Females aged more than 45 years and those with acromegaly, hypothyroidism, hyperprolactinemia, non-classic congenital adrenal hyperplasia, metabolic syndromes, or Cushing’s syndrome were excluded from the study.

### Ethical Approval:

The Aga Khan University Ethical Review Committee approved the study, and the rights of the participants were protected (ERC AKU 2024-9121-28391, dated: March 15, 2024). Non-probability purposive sampling method was employed and sample size of 99; 49 PCOS (cases) and 50 (control) subjects was determined by the formula:


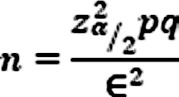


Where *n* is the required sample size, *zα⁄2* is the Z-score for the desired confidence level (e.g., 1.96 for 95%), *p* is the expected population proportion, *q = 1 - p* is the proportion without the characteristic, and *∈* (epsilon) is the margin of error or precision level (e.g., 0.05 for 5%). It included a non-probability purposive sampling method of selecting participants whose criterions of inclusion as PCOS (cases) and those without any PCOS characteristics (controls) were strictly followed. Although this is reducing generalizability, it makes testing of genotype-hormone associations more homogeneous and clinically pertinent with less heterogeneity.

Standard data collection proforma included: sociodemographic information, age, marital status, family history, medical history, height, weight and hormone profile. Four ml blood was collected in two vacutainers for biochemical (gel vacutainer) and genetic analysis (EDTA). Serum was separated from whole blood by centrifugation (5000 rpm/ 5min), AMH levels were assessed using a commercial immunoassay kit (BT LAB ELISA Kit, Cat. No. E1052Hu). DNA was isolated by QUAIGEN genomic DNA extraction Kit and stored at 4°C.

The UCSC Genome Browser at the University of California, Santa Cruz (UCSC) was utilized to obtain the *AMH* (rs10407022) sequence. Tetra-Arms Primers were designed for the rs10407022 by freely available online software Primer1 (http://primer1.soton.ac.uk/primer1.html) Dated 11 March 2024. The obtained primer by Primer1 were further optimized by considering GC content, heterodimer, melting temperature, homodimer, hairpin loop, etc. by online tool OligoAnalyzer (https://eu.idtdna.com/pages/tools/oligoanalyzer). An in-silico amplification of rs10407022 by T-ARMS-PCR was checked by in silico PCR tool of UCSC genome browser (https://genome.ucsc.edu/cgi-bin/hgPcr).

PCR reaction condition for genotyping of rs10407022, PCR mixture reaction of final volume 10 μL were prepared for each sample/PCR reaction. For PCR mixture single reaction 50-100 ng of DNA was used. DNA polymerase used 2x PCR Thermos Scientific PCR Master Mix (Cat# K0171, ABM (Applied Biological Materials Inc, Canada). The initial denaturation was at 94°C for three minutes, then denaturation 94°C for 30 seconds, annealing time was 65°C for 30s, extension at 72°C for 45 seconds for 35 cycles and final extension at 72°C for 10 minutes. The PCR products were then visualized using the Gel Electrophoresis technique on an agarose gel stained with 1% ethidium bromide with 100 bp ladder (cat no. 2817945) on Biorad Chemi Doc imaging equipment. The use of ethidium bromide was carried out in a careful manner because this agent is mutagenic. Any manipulations were conducted using gloves and lab coats, in restricted biosafety locations and EtBr waste was eliminated according to the biosafety standards of institution. For validation, genotyping was further validated by the Polymerase Chain Reaction-Restricted Fragment Length Polymorphism (PCR-RFLP). For PCR-RFLP the *AMH* DNA sequence recovered by in-silico PCR was utilized to determine the restriction site of the amplified target sequence. The restriction sites were located using the web application NEBcutter V2.0. http://NEBcutter2/nc2.neb.com. The outer primers were used to amplify the target site of SNP rs10407022 and the amplified sequenced were digested by: BtsCI Cat No.ER0871 enzyme from Thermo Scientific, Waltham, MA USA.

Digestion of the amplified DNA sequences for SNPs rs10407022 was finally completed with an Eppendorf PCR-Thermal Cycler. For complete digestion, the reaction mixture in the PCR plates were incubated for 180 minutes at 37°C. After incubation, the enzyme was denatured by heating the plate for five minutes at 85°C (Restriction Enzyme; 0.5 μL, 10x Buffer; 1 μL, PCR Product; 3 μL, ddH2O; 5.5 μL, Total Volume; 10 μL). Following digestion, a 2% agarose gel stained with ethidium bromide was loaded with the PCR-RFLP product with 100 bp DNA ladder (cat no. # 2817945) visualized by ChemiDoc Imaging System and finally the PCR-RFLP products DNA bands were examined for genotyping. For validation purposes, gold standard DNA Sanger sequencing was performed on different random samples of cases (PCOS) from both assays. The random samples for Sanger DNA sequencing were selected based on the Homo wild type (WT) G/G, mutated T/T, and heterozygous alleles G/T.

### Statistical analysis:

SPSS version 20 software was used for all statistical analysis. A p-value of less than 0.05 was considered significant.

## RESULTS

Results show that PCOS group had a significantly lower mean age compared to the non-PCOS group (28.43 ± 5.63 years vs. 30.36 ± 7 years; p-value of 0.03). AMH levels were markedly higher in the PCOS group (6.47 ± 6.05) than in the non-PCOS group (3.06 ± 1.91); p-value of 0.0001. Prolactin levels were also significantly elevated in PCOS group (19.34 ± 10.27) compared to non-PCOS group (14.3 ± 7.15), with a p-value of 0.05. No significant differences were found between the two groups for Body Mass Index (BMI): p = 0.57, Luteinizing Hormone (LH): p = 0.72, Thyroid-Stimulating Hormone (TSH): p = 0.28, Follicle-Stimulating Hormone (FSH): p = 0.98 (Table-I).

**Table-I:** Comparison of Study Variables in Cases (PCOS) and Controls.

	PCOS (n=49)	Non-PCOS (n=50)	p-value
Age (years)	28.43 ± 5.63	30.36 ± 7	0.03
BMI (kg/m2)	26.53 ± 7.01	28.71 ± 6.11	0.57
AMH (ng/mL)	6.47 ± 6.05	3.06 ± 1.91	0.0001
Luteinizing Hormone LH (mIU/L)	7.5 ± 3.18	5.81 ± 3.03	0.72
Thyroid-Stimulating Hormone TSH (mIU/L)	2.2 ± 1.57	1.91 ± 1.13	0.28
Follicle-Stimulating Hormone FSH (mIU/mL)	7.38 ± 6.54	7.22 ± 3.43	0.98
Prolactin (μg/L)	19.34 ± 10.27	14.3 ± 7.15	0.05
Thyroxine T4 (mcg/dL)	8.9 ± 2.18	8.44 ± 2.53	0.58

Independent T Test

### ARMS-PCR Results:

**Fig.1:**
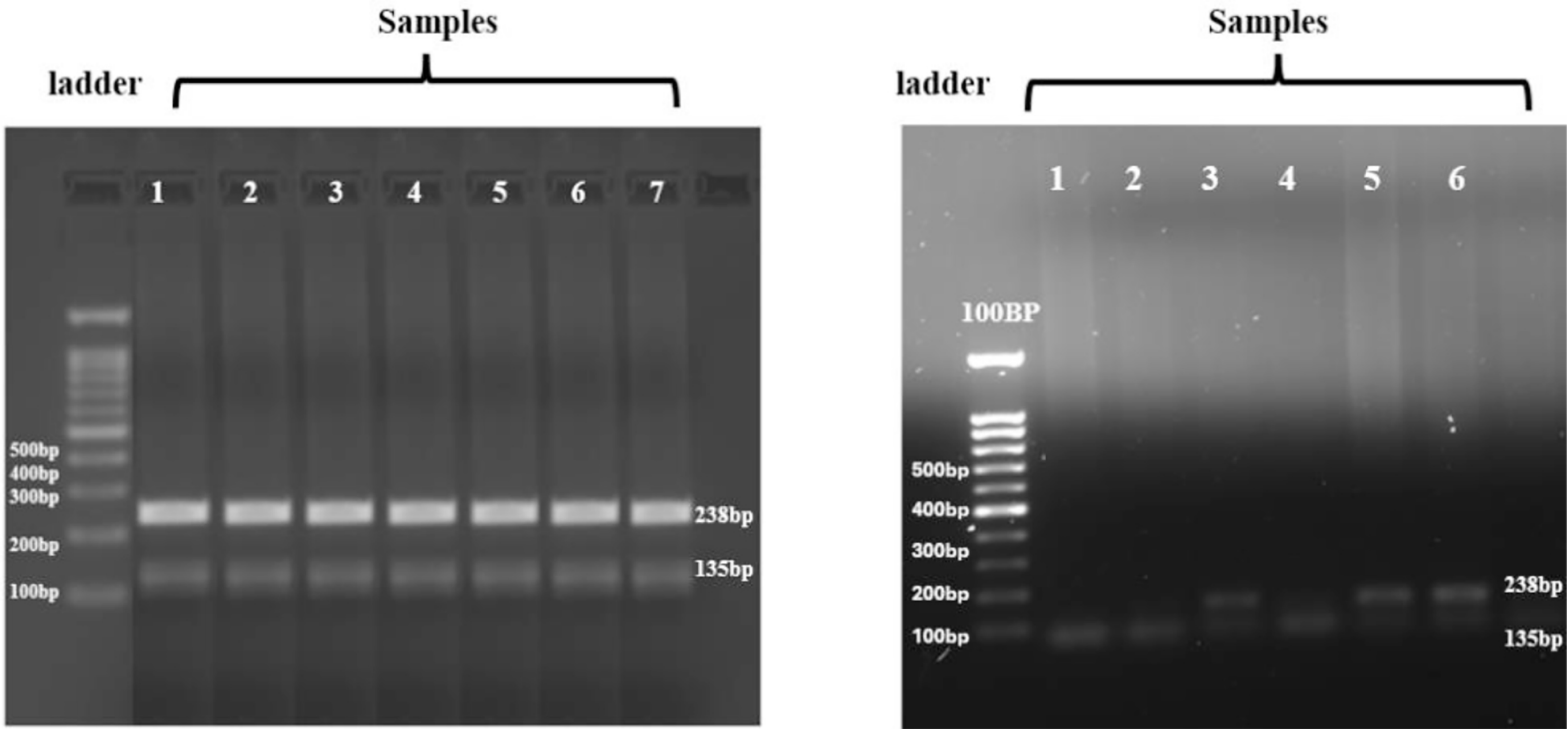
Agarose gel electrophoresis of PCR-amplified AMH gene (rs10407022). (a)DNA ladder 100 bp, L1-L5 present PCR amplified product from case samples(b). DNA ladder 100 bp, L1-L5 present PCR amplified product from control samples All the PCR amplified product show expected band size.

The results of PCR amplification for the *AMH* (rs10407022) in both PCOS and non-PCOS groups are shown in (Fig.1). Lanes one to five represent the amplified DNA fragments of PCOS cases, with a 100 bp DNA Ladder as a reference [Fig.1(a)]. No bands were observed in the non-PCOS group, showing the absence of the preferred gene in controls at the required band size of 135bp and 238bp [Fig.1(b)]. These gel electropherograms successfully confirmed the amplification and validated the use of PCR for genotyping.

### Restriction fragment length polymorphism (RFLP) Results:

**Fig.2:**
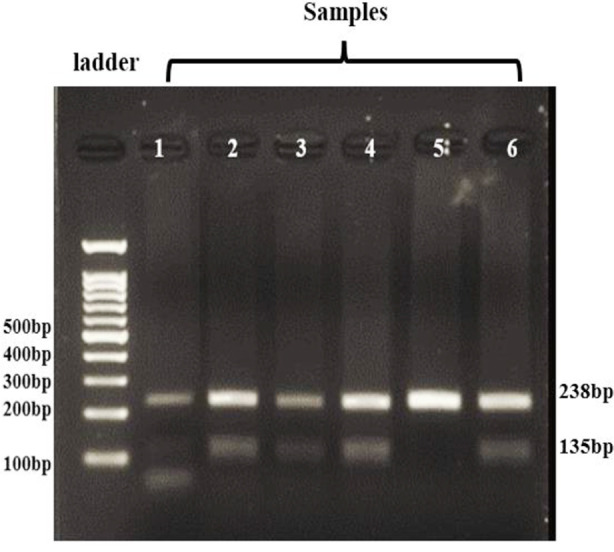
Gel electropherograms of AMH PCR-restriction fragment length polymorphism (RFLP) product results for rs10407022. DNA ladder 100 bp, L1-L6 present PCR-RFLP product. Each lane represents a distinct genotype based on PCR-RFLP.

The RFLP results for *AMH* gene polymorphism (rs10407022) illustrated in Fig.2 clearly demonstrate the digestion patterns of amplified DNA fragments, with a 100 bp plus DNA Ladder serving as a size marker. These results confirm the efficiency of restriction enzyme digestion and the differentiation of genotypes.

**Fig.3:**
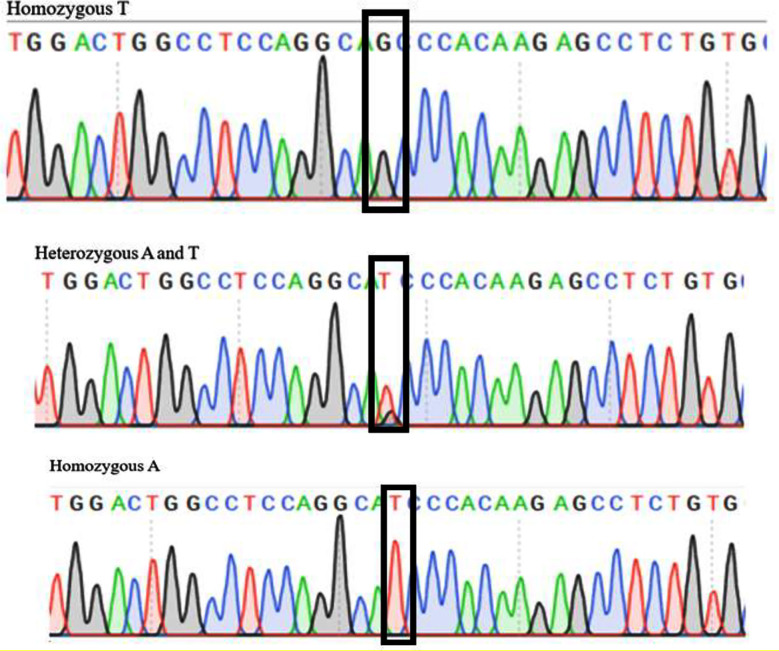
DNA sequencing electropherograms of *AMH* PCR-RFLP result product for **(a)** Homozygous wild-type genotype (**GG**); **(b)** Heterozygous genotype (**GT**); **(c)** Homozygous mutant genotype (**TT**).

Sanger DNA sequencing was performed for verification of insertion of mutation in *AMH* (Fig.3). For sequencing, each tube contained 75ng of purified PCR product. The chromatograms generated from sequencing exhibited distinct peaks corresponding to the nucleotide sequences of the *AMH* (rs10407022) gene. This confirmation underscores the reliability and accuracy of the genotyping results presented in this study.

## DISCUSSION

In our study, the significant difference in AMH levels between the two groups advocates its role as a biomarker for PCOS diagnosis and monitoring.^18^ Furthermore the AMH levels were more than the cut off levels (3.8–5 ng/mL) as suggested for diagnosis of PCO.^12^ The significant increase in prolactin levels observed in our study supports the role of elevated prolactin in PCOS, which has been shown to disrupt the hypothalamic-pituitary-ovarian axis, potentially exacerbating symptoms such as menstrual irregularities and infertility.^19^

There are several genes involved in steroidogenesis, obesity, inflammation, gonadotropin action, insulin production, and resistance which contribute to the pathogenesis of PCOS.^20^ In reproductive-age women from Punjab, Pakistan, a significant association was found between PCOS susceptibility and polymorphisms in CYP*17* (rs743572) and *CYP19* (rs2414096).^21^ Similarly, the *ERBB4* variant (rs2178575) was found to decrease the risk of infertility in females having PCOS in selected population of Karachi.^22^ This study provides further evidence that specific genetic variations in *AMH* are involved in the pathogenesis of PCOS.

Lack of bands in the non-PCOS (control) group by ARMS PCR indicates that the genetic variant (rs10407022) is either absent or not amplified in this group, reinforcing the distinct genetic profiles of PCOS and non-PCOS groups. The observed variations in fragment sizes following restriction enzyme digestion confirmed the presence of different alleles in the PCOS and non-PCOS groups. The clear banding patterns in our study demonstrate the efficiency and specificity of this technique demonstrating the technique’s usefulness in distinguishing between genotypes. This differentiation supports the hypothesis that genetic variations in the *AMH* contribute to the pathophysiology of PCOS.^20^ Sanger sequencing, known for its high sensitivity and accuracy^23^ enabled the precise detection of the rs10407022 mutation and demonstrated its potential as a reliable tool for genetic analysis in PCOS research.

The presence of both wild type and mutated alleles in our samples is particularly relevant, as it reflects the genetic variability associated with this polymorphism. The ability to differentiate between these genotypes is crucial for understanding the genetic diversity within the population of women with PCOS and could provide insights into the role of specific alleles in the development of the disorder. Our study has shown significant difference in genotype frequencies of the rs10407022 polymorphism between women with PCOS and matched controls, suggesting a possible association between this SNP and PCOS susceptibility.

### Strength of our study:

One of the strengths of this study is the use of well-defined diagnostic criteria for PCOS along with BMI-matched controls, which allowed for a more accurate comparison of genotype distributions. The study also focused on a gene previously linked to PCOS, enabling a more targeted investigation of a biologically relevant SNP. Furthermore, the genetic variant was identified using tetra-primer ARMS-PCR, validated through RFLP, and confirmed by Sanger sequencing, ensuring the reliability of the genotyping results.

### Limitations:

A key limitation of our study is its focus on a single nucleotide polymorphisms (SNPs). Given the heterogeneity of PCOS and to better uncover its genetic causes, Whole Genome Sequencing (WGS) should be considered in future studies to explore both coding and non-coding regions of the genome.^24^ Since non-probability purposive sampling technique was applied, it is possible that the results cannot be applied to the wider population. Nonetheless, this recruitment method made it possible to carry out selective recruitment of clinically characterized cases and controls which enhances internal validity of our comparisons. Moreover, as this is a small-scale preliminary study, it should be expanded to include a larger sample size with ethnically diverse cohorts. This would help identify additional genetic variants and allow for assessment of the functional impact of this gene variation on AMH levels. Such insights could strengthen its potential as a biomarker for PCOS diagnosis and risk prediction, ultimately contributing to improved women’s health outcomes.

## CONCLUSION

High levels of AMH and genetic variant (rs10407022) detected by Tetra Arms PCR and RFLP further validated by sequencing suggest the role of dysregulation of *AMH* in the manifestation of PCOS. Further research is needed to understand the clinical implications and explore the use of genetic markers in practice.

***Disclaimer:*** i) The manuscript is part of MPhil thesis of Ms. Laiba Rehmat ii) AI-Chatbots were used in the preparation of the manuscripts, to improve readability and language.

### Authors’ Contribution:

**LR:** Executed the MPhil project; performed bench work, analyzed the data and wrote the results

**RR:** Designed and supervised the study.

**SZ, UK and HNK:** Co-supervised the whole project from data collection, bench work, data analysis and write up of thesis.

All authors took part in the write-up of the manuscript, read and revised the content and all are accountable for the accuracy or integrity of the work.

## References

[1] Huffman AM, Rezq S, Basnet J, Romero DG (2023). Biomarkers in polycystic ovary syndrome. Curr Opin Physiol.

[2] Azhar A, Abid F, Rehman R (2020). Polycystic ovary syndrome, subfertility and vitamin D deficiency. J Coll Physicians Surg Pak.

[3] Jiang B (2025). The Global Burden of Polycystic Ovary Syndrome in Women of Reproductive Age:Findings from the GBD 2019 Study. Int J Womens Health.

[4] Luo J, Li Z, Wang Z, Ding Y, Gao P, Li Y (2025). Efficacy of probiotics combined with metformin and a calorie-restricted diet in obese patients with polycystic ovary syndrome. Pak J Med Sci.

[5] Rafique A, Salma UE, Saleem HG (2023). Measuring the awareness of polycystic ovarian syndrome (PCOS) among women in Punjab, Pakistan. Sci Inquiry Rev.

[6] Li X, Tang Q, Feng Y, Zhang Y, Tian W, Zhang H (2024). The Relationship Between Anti-Müllerian Hormone and Body Composition Components in Patients with Polycystic Ovary Syndrome of Reproductive Age. Reprod Sci.

[7] Koyama T, Suzuki H, Shimizu M, Mizuno R, Ishigami A, Kamidate N (2024). Assessment of anti-Müllerian hormone levels as a reproductive indicator in Japanese Black cattle. J Reprod Dev.

[8] Aalpona FZ, Ananya KF, Kamrul-Hasan AB (2023). Correlation of Serum Anti-Mullerian Hormone with Clinical, Metabolic and Hormonal Parameters in Bangladeshi Women with Polycystic Ovary Syndrome:A Cross-sectional Study. Mymensingh Med J.

[9] Khan MF, Parveen S, Sultana M, Zhu P, Xu Y, Safdar A (2024). Evolution and Comparative Genomics of the Transforming Growth Factor-β-Related Proteins in Nile Tilapia. Mol Biotechnol.

[10] Munira S, Banu J, Ishrat S, Shume MM, Uddin MJ, Sultana S (2021). Anti-Mullerian Hormone (AMH) as a Predictor of Ovarian Response to Clomiphene Citrate in Polycystic Ovarian Syndrome. Fertil Reprod.

[11] Büyükyılmaz G, Koca SB, Toksoy Adıgüzel K, Boyraz M, Gurbuz F (2024). The Role of the AMH, SHBG, Free Androgen Index and LH/FSH Ratio in the Diagnosis of Polycystic Ovary Syndrome in Adolescent. Turkish J Pediatr Dis.

[12] Butt MS, Saleem J, Aiman S, Zakar R, Sadique I, Fischer F (2022). Serum anti-Müllerian hormone as a predictor of polycystic ovarian syndrome among women of reproductive age. BMC Womens Health.

[13] Dewailly D (2016). Diagnostic criteria for PCOS:is there a need for a rethink?. Best Pract Res Clin Obstet Gynaecol.

[14] Lie Fong S, Laven J, Duhamel A, Dewailly D (2017). Polycystic ovarian morphology and the diagnosis of polycystic ovary syndrome:redefining threshold levels for follicle count and serum anti-Müllerian hormone using cluster analysis. Hum Reprod.

[15] Chen D, Zhu X, Wu J (2020). Can polymorphisms of AMH/AMHR2 affect ovarian stimulation outcomes?A systematic review and meta-analysis. J Ovarian Res.

[16] Chaudhary H, Patel J, Jain NK, Panchal S, Laddha N, Joshi R (2024). Investigating the interplay between AMH gene polymorphism rs10407022 and clinical indicators in polycystic ovary syndrome. Human Gene.

[17] Dokuru DR, Horwitz TB, Freis SM, Stallings MC, Ehringer MA (2024). South Asia:The Missing Diverse in Diversity. Behav Genet.

[18] Bhattacharya K, Saha I, Sen D, Bose C, Chaudhuri GR, Dutta S (2022). Role of anti-Mullerian hormone in polycystic ovary syndrome. Middle East Fertil Soc J.

[19] Mahboobifard F, Rahmati M, Amiri M, Azizi F, Tehrani FR (2022). To what extent does polycystic ovary syndrome influence the cut-off value of prolactin?Findings of a community-based study. Adv Med Sci.

[20] Bhimwal T, Puneet, Priyadarshani A (2023). Understanding polycystic ovary syndrome in light of associated key genes. Egypt. J Med Hum Genet.

[21] Munawar Lone N, Babar S, Sultan S, Malik S, Nazeer K, Riaz S (2021). Association of the CYP17 and CYP19 gene polymorphisms in women with polycystic ovary syndrome from Punjab, Pakistan. Gynecol. Endocrinol.

[22] Samma ZH, Khan HN, Riffat S, Ashraf M, Rehman R (2024). Unraveling the Genetic Associations of DENND1A (rs9696009) and ERBB4 (rs2178575) with Infertile Polycystic Ovary Syndrome Females in Pakistan. Biochem Genet.

[23] Cheng C, Fei Z, Xiao P (2023). Methods to improve the accuracy of next-generation sequencing. Front. bioeng. Biotechnol.

[24] Khan MJ, Nazli R, Ahmed J, Basit S (2018). Whole Genome Sequencing instead of Whole Exome Sequencing is required to identify the Genetic Causes of Polycystic Ovary Syndrome in Pakistani families. Pak J Med Sci.

